# Water—Some of Its Physiological and Therapeutical Uses

**Published:** 1912-09

**Authors:** J. H. Neall

**Affiliations:** Atlanta, Ga.


### WATER—SOME OF ITS PHYSIOLOGICAL AND THERAPEUTICAL USES.

By J. H. Neall, M. D., Atlanta, Ga

As far back as 1799, a man was born at Grafenberg, Germany, who was destined to make popular the use of natural remedies in the treatment of disease. This man was Vincent Priessnitz. He had few ducational advantages. Of his early life we know very little, and of his later work we know nothing except through the writings of others. At the age of thirteen he sprained his wrist. While attending to his father's cattle, he closely observed their instincts, and also those of many of the wild animals, which were numerous in that region. He observed a deer specially, which had injured her leg. She limped for several successive days to a mountain stream and drank of the water and stood in it for a long time. Thinking about the circumstances, he thought of the idea of holding his painful wrist under the pump. This, he found, gave him great relief. He then hit on the plan of applying a cold compress to his wrist, changing it every time it became warm. The wrist was quickly restored, and he was so pleased with the result that he wanted to try it on others. Another instance: at the age of sixteen he met with a severe accident. He had two ribs broken, and was covered with bruises. Local physicians gave him no hope of recovery. He remembered the cold application he had used before, and at once applied the same applications by means of the cloths kept wet in cold water to the injured parts, and quickly relieved the inflammation. Then, standing up and pressing his abdomen against the window sill, and inflating his lungs with all his might, he succeeded, in this crude manner, in setting his own ribs. In a short time he was cured completely.

Priessnitz never read a book on medicine. Yet he had such penetrating observation that the people who consulted him almost believed that he could look into their bodies as if they were made of glass. At a glance he noted the appearance of the patient's skin, his countenance, his general demeanor and movements, his voice, and especially his eyes. He was always on hand to see how the patient responded to the first treatment, and his prescription almost invariably had the effect he expected. It is said that he never lost a fever case.

The marvelous success of this nature-taught man attracted the attention of even the crowned heads of Europe. The French government sent the head of the Medical Department of the Army to study his methods, and by this means hydro-therapy was introduced into the military service of France. The governments of other countries did likewise. Thus the principles and methods, developed and systematized by this man were rapidly diffused. To-day they form the basis of modern hydro-therapy.

When Providence would bless humanity, the man is raised up to blaze the way through untried fields to present new principles in the healing art. The application of natural remedies made popular by such men as Winternitz in Germany, Fleury in France, and Currie, John Bell, Baruch, and Kellogg in this country, is no longer to be considered as a fad, but as a legitimate part of rational therapeutics. The scientific application of water should be a part of every real physician's practice.

I quote from Kellog, that "Among the Spartans of ancient Greece, cold bathing was made obligatory by law. The bath in various forms is also frequently referred to in Grecian mythology. Hippocrates evidently understood the physiological properties of water, both hot and cold, which he employed in the treatment of fevers, ulcers, hemorrhages and a variety of maladies, both medical and surgical, giving many directions for its use which the experience of two thousand years has not improved upon. For instance, he directed that cold baths should be of short duration, and should be preceded and followed by friction; and he evidently understood the phenomna of reaction, since he records the observation that after a cold bath the body recuperates the heat and remains warm, while a hot bath produces the opposite effect. Asclepiades employed water in nearly every form, hot and cold baths, douches, compresses, etc. According to Pliny, the bath was almost the exclusive method of treatment employed in Rome during five centuries. Celsus, and other prominent Roman physicians highly extolled the bath in their work. Celsus made it one of the three essentials of what he called a perfect therapeutic system, termed "apotheraphia," the other two being exercise and friction. During the middle ages, the Arabian physicians, the most learned men of their time, were enthusiastic advocates of the bath, especially in fevers, and their directions for the treatment of small-pox and measles could scarcely be improved upon at the present time. Rhases recommended drinking ice water to the extent of two or three pints within half an hour as a means of reducing the temperature in fever. Avicenna recommended cold water for the relief of constipation."

Who does not read with abhorrence, and blush with shame, at the suffering caused by the denial of water to patients burning up with fever? Almost within our own memory we can remember when it was the practice to deny to typhoid patients the free drinking of pure water. Now, no up-to-date physician would think of taking his patients through the typhoid disease without making use of applications of water in some form or other.

Who has not read with pride and satisfaction of the successful application of the Brand bath, and the lower percentage of deaths from typhoid fever following its use?

It will be quite impossible, in the time allotted to us in the reading of our paper, to go into minute details, but a few therapeutic uses of water may be of interest and help at this point.

When it is considered that all our functions are performed, as it were, under water, we can form some faint idea of how necessary it is to us physiologically. The intense suffering of the individual deprived of water, and the ensuing insanity if thirst is not relieved, is a striking illustration of its importance to the human economy. At least one liter and a half, a little over three pints, should be ingested in the run of a day. Not only is it necessary that water should be drunk in sufficient quantity, but, being the one universal solvent, it should be applied externally to the whole surface of the body, at least once a day. This may be done by means of a cold sponge bath, hot and cold shower, and once a week a hot tub bath with soap to remove the accumulated oil upon the skin.

Old Dr. Abernathy it is said, once had a patient that manifestly needed to have the accumulations on the skin removed in order that part of her anatomy might perform its function. In giving his prescription, he told her to fill a tub partly full of water at a certain temperature, and with the use of soap, brush and towels, remove as much of the outside covering as possible. "Isn't that somewhat in the nature of a bath?" asked the lady. "Well, madam," replied the good doctor, "It *is* open to that objection." There are many even in our time who would be benefited by such a prescription.

"The person who wishes to keep well and look well should cultivate a belief in water. Every woman who values her complexion, should drink six glasses of cold water a day. If desired, the glass taken before retiring, and the first one in the morning may be hot, with a pinch of salt in it. Have regular hours for taking water. The periods may be divided in the following manner: in the morning, as soon as you arise; the last thing before retiring at night; half an hour before luncheon, and dinner; and in the middle of the morning and afternoon."

Water is a fairly good conducter of heat. Its conductivity is much greater than air, but less than that of metals. Copper conducts heat one hundred times better than water. A much more intense impression is made by water at any given temperature, hot or cold, upon the skin, than is made by air at the same temperature.

Ordinary water is a good conductor of electricity. It is valuable in combined procedures, such as hydro-faradic, hydro-galvanic, and similar baths. In the body, water is the medium by which the foods, rendered soluble by digestion, and conveyed to the tissues to be assimilated, and thus rendered insoluble, when the effete matters, rendered soluble by disassimilation, are dissolved and conveyer back into the blood-current to be acted upon or eliminated by the liver, the kidneys, skin and other excretory organs. The two chief constituents of digested food, sugar and peptones are among the most soluble of substances. Urea, a product of proteid oxidation, has a high degree of solubility in water. Uric acid and oxalic acid, and other abnormal products are, on the other hand, less easily soluble than the normal waste products, and hence, as has been shown by Haig, readily accumulate in the body, especially in those portions where the circulation is least active.

The science and art of hydro-therapy, or the use of water as a curative agent, include not only applications of water in its various forms, but thermic applications made by means of hot or cold air, vapor baths, various heated objects, and also heat and light. One of the very best means for promoting elimination is found in the electric light bath cabinet, which is a cabinet in which the incandescent lamp is utilized as the source of heat. The cabinet is lined with mirrors on all sides, which increases the intensity of the heat by reflection. In a paper read before the American Electro-therapeutic Association, at its meeting in New York, in September, 1894, Dr. J. H. Kellogg gives details of many experiments which had been conducted for the purpose of determining the physiological effects of this bath. The general conclusions resulting from his investigations were as follows:

"1. The electric-light bath stimulates the elimination of $CO_2$ in a very marked degree. In an electric-light bath lasting 30 minutes the percentage of $CO_2$ elimination during the last ten minutes was 5.13, as compared with 3.60, the average of $CO_2$ elimination before the bath. In a Russian bath the percentage of elimination was 3.96. In a Turkish bath of 30 minutes' duration, the percentage of $CO_2$ elimination was 4.01, while the elimination of urea was diminished.

"2. The elimination of nitrogenous wastes represented by urea, and also the elimination of total solids, was greatest in the Russian bath, and least in the electric-light bath.

"3. The amount of perspiration produced by the electric-light bath was fully double that induced by the Turkish bath in the same length of time. The time required for the first appearance of perspiration was, in the electric-light bath, about one-half the time of that in the Turkish baths. The reason for this is apparent when one recalls the fact observed by Bouchard, that perspiration begins when the temperature of the blood has risen .7 degree above the normal. In the study of the physiological effects of the electric-light bath in 1891, when the author made his first experiments upon this subject, he found the internal temperature was raised by the bath 1.6 degrees in five and one-half minutes, the surface temperature rising, in the same time, 2.3 degrees F."

Following the electric-light bath, the shampoo, the sprays, and showers make a very delightful and useful treatment in many cases of disease. Hot and cold water by means of a mixer, wholly under the operator's control, and a thermometer showing the temperature at any and all stages, with a good pressure renders excellent service after the electric-light bath.

A very efficient way of applying heat by means of water locally is by the fomentation, which consists of two pieces of flannel, each the size of a quarter of a blanket. One piece is to remain dry, and the other is to be wrung out in boiling water. The dry flannel is to be laid on the part to be treated, and over this applied the wet flannel, folded to the required size. This should be done three times, either alternating with cold or ending with cold, in the form of water or ice.

Another method of applying local heat is by means of a photophore, which is an appliance containing one, two or three incandescent lights. This can be made to produce a moist or dry heat by direct application, or by the intervention of moist cloths between it and the body.

Cold compresses for the application of cold locally will be found most helpful in a case such as typhoid fever, or inflammations of the internal organs. The compress, to be effectual, should be frequently changed, interrupting at intervals of two or three hours with short fomentations. This becomes necessary on account of the continued application of cold, destroying the sensation, and eliminating the reflex effects.

The application of water by means of the sponge bath carries with it much comfort to the patient, and a lowering of the temperature. This, to be effectual, must be applied scientifically.

In a case of rising temperature, the application of cold will, on account of the reaction, tend to cause a greater rise after it. Hence, to avoid the reaction, the sponging should be done with warm water, and in such a way that the evaporation will lower the temperature, thus avoiding a reaction. This is very important, as by the other means you will add to the discomfort of the patient, the automatic heat centers being prompted to activity by any sensation of cold. The sponge bath is especially useful in a case of fever, but it should be emphasized that care should be taken by the nurse in a fever case to see that the patient's feet and limbs are kept warm. Any sensation of cold is a signal to the heat centers to manufacture more heat.

The use of the normal saline solution by enema at a temperature of 110 degrees to 120 degrees in a case of shock will be found one of the most effectual means at our command to relieve that dread condition. The application of cold continuously applied to the region of the heart will also be found a most valuable therapeutic adjunct in such a case. By this latter means the heart is slowed down, and its strength increased.

The attempt of some to cure constipation by means of large enemas used continuously can not be too strenuously deprecated. The continual distention of the lower bowel by large quantities of water is anything but a desirable therapeutic procedure. In place of this, the graduated enema is much to be preferred. This consists in giving a high, thorough enema at a moderate high temperature, and gradually diminish the quantity and lower the temperature until a very small amount of water is used at a low temperature, stimulating peristaltic action rather by means of the cold than by means of the quantity. This has been found to work admirably in the experience of your essayist.

Nothing can be more desirable than the popularizing of the use of such simple remedies in the place of many patent medicines containing so much that is detrimental to their users. A learning to use water judiciously will accomplish far more for the lay people without injury, or the contraction of a habit, than any or all of the most desirable patent medicines upon the market.

Authorities quoted: Dr. J. H. Kellogg, in "Modern Hydrotherapy;" Edgar Brigham.

236 Gordon St.

